# Extracorporeal cardiopulmonary resuscitation in 2023

**DOI:** 10.1186/s40635-023-00558-8

**Published:** 2023-10-30

**Authors:** Tobias Wengenmayer, Eike Tigges, Dawid L. Staudacher

**Affiliations:** 1 Interdisciplinary Medical Intensive Care, Faculty of Medicine and Medical Center-University of Freiburg, Hugstetterstrasse 55, 79106 Freiburg, Germany; 2 Department of Cardiology and Critical Care, Asklepios Clinic St. Georg, Hamburg, Germany

## Introduction

Survival rates after cardiac arrest are low. In this context, extracorporeal cardiopulmonary resuscitation (ECPR) has been first introduced as ultima ratio in 1966 [1]. Almost 6 decades later, more than 15,000 ECPR cases are registered in the Extracorporeal Life Support Organization (ELSO) registry [2, 3] with a survival rate of 30%. This review will discuss the background, indications, challenges, and limitations of ECPR.

## Rationale for ECPR

Approximately 8% of individuals who experience out-of-hospital cardiac arrest (OHCA) survive, with significant variations observed between different countries and cohorts, ranging from 0 to 18%. Survival rates between 15 and 34% have been reported for in-hospital cardiac arrest (IHCA) [4]. One of the dominant predictors of survival is the delay between arrest and return of spontaneous circulation. After 15–20 min of conventional cardiopulmonary resuscitation (CCPR), the probability of survival with good neurological function is approximately 2% [5]. CCPR achieves a mere 25% to 30% of native cardiac output [6] leading to a progressive tissue hypoxia and ultimately death, which might contribute to this outcome. The duration of CCPR with insufficient circulation is coined low-flow duration [7]. The rapid restoration of perfusion and oxygen supply to vital organs plays a crucial role in the chain of survival and the quality of life of patients after cardiac arrest [4, 8]. ECPR ensures sufficient organ perfusion, including the brain, in patients without ROSC [8]. After the progressive detonation of prognosis during low-flow is resolved, the cause of collapse can be resolved.

## Definitions

ECMO (extracorporeal membrane oxygenation) or ECLS (extracorporeal life support) are used synonymously in literature [9]. According to the cannulation, two main operational modes of ECMO are used: the venovenous (V-V) mode for pulmonary failure and the venoarterial (V-A) mode for circulatory failure or ECPR [10]. This review will exclusively cover V-A ECMO in the context of ECPR.

ECPR is defined as V-A ECMO cannulation during refractory cardiac arrest [11]. Refractory cardiac arrest is considered as the absence of ROSC despite provision of appropriate CPR for 15 [12] to 30 min [13]. Moreover, ECPR includes patients with on/off CPR and those without stable ROSC. Since a stable ROSC is defined as spontaneous circulation for at least 20 min without chest compressions and persistent circulation [14], also V-A ECMO cannulations within 20 min after ROSC is considered ECPR [11]. Unfortunately, the ECPR definition does not specify which part of the V-A ECMO cannulation has to be completed within 20 min after a potentially stable ROSC in order to still qualify for ECPR (only the intention to cannulate, successful puncture of a vessel, placement of both cannulae, or a running ECMO system). Theoretically, there might be a big difference in disease severity and outcome between a patient with potential stable ROSC (that just did not meet the 20 min criteria) and a patient with truly refractory arrest. Insufficient clarity in defining ECPR hinders the comparability of scientific studies, introducing potential biases. According to the Utstein definition, placement of a venovenous ECMO (V-V ECMO) during CPR (in primary pulmonary failure) is not considered ECPR [14].

## ECMO system

The ECMO system consists out of six integral components: cannulae for vascular access, lines, a pump, an oxygenator, a heat exchanger and an interface [15]. Many different cannulae are commercially available which differ in handling and technical features [16]. For V-A ECMO, typical draining cannulae range from 21 to 27 Fr., while returning cannulae are smaller and shorter (15 to 19 Fr.) [17]. The draining cannula is connected to the pump. For adults, mostly centrifugal pumps are used to circulate the patient's blood through the ECMO circuit and to provide the pressure necessary to maintain a constant blood flow throughout the ECMO system and back to the patient [18]. Next in line is the oxygenator. The oxygenator consists of a semipermeable membrane that allows gas exchange to occur. This core component of the ECMO circuit is responsible for enriching oxygen and reducing carbon dioxide in the patient’s blood [19]. Last part of the V-A ECMO circuit is the arterial (returning) cannula. In order to avoid accidental hypothermia and for targeted temperature management, a heat exchanger is connected to the circuit. Finally, the ECMO is controlled by an interface to provide feedback to the healthcare team. The oxygenator mimics the lung’s gas exchange function, while the pump provides up to 6 l/min blood flow. The maximum blood flow is contingent upon the choice of cannulae, where smaller cannulae impose higher resistance resulting in heightened shear stress and lower ECMO flow. The transfer of oxygen and carbon dioxide within the oxygenator can tailored to patient needs by adjusting the sweep gas flow and its oxygen concentration. In V-A mode, ECMO can functionally approximate full cardiopulmonary bypass by providing retrograde mechanical circulatory support.

## Cannulation

In general two cannulation techniques are employed, central and peripheral cannulation [20]. For central cannulation, a sternotomy is required in order to place the cannulae directly in the right atrium and the ascending aorta [20]. Compared to this highly invasive approach, peripheral cannulation is far less invasive. In literature, similar neurological outcomes [20–22], mortality [23, 24], and peripheral ischemia [24, 25] were reported comparing central and peripheral cannulations while bleeding complications were more frequent in central cannulation [24–26]. Since peripheral cannulation does not require sternotomy, is more readily available and thus practically the only applied technique in an ECPR scenario [27, 28]. In the three large randomized controlled ECPR trials, peripheral cannulation was used [12, 29, 30]. Specifically, large-bore cannulas are introduced through the patient’s groin vessels (common femoral vein and common femoral artery), with their tips positioned in the superior vena cava and either the aorta or common iliac artery, respectively. The procedure is commonly performed percutaneously under sonographic guidance using the Seldinger’s technique [31]. In some cases, fluoroscopy may be employed for further assistance, particularly when the procedure is conducted in the cardiac catheterization laboratory [32, 33]. A surgical cut down is also an option [34], usually not used as first-line approach. To drain venous blood, a 21 to 27 French cannula (inner diameter 7.0 to 8.9 mm), ideally extended to the superior vena cava, is utilized [35]. This cannula has a large opening at its distal end and numerous side openings, allowing it to draw blood from a considerable portion of the upper and lower vena cava as well as from the right atrium of the patient. The returning, arterial cannula typically has 15 to 19 French (inner diameter 5.0 to 6.3 mm) and returns the blood to the patient retrograde. The entire cannulation and connecting process takes approximately 10–15 min for experienced teams [29, 30].

## Patient selection

Identifying the appropriate candidates for ECPR is complex [11, 36–38]. Factors including witnessed collapse, bystander CPR, initial rhythm, medical conditions, and biological age (correlated with life expectancy) have been discussed [39–43]. At the present time, there is still ambiguity regarding clear inclusion and exclusion criteria. National recommendations differ, and prospectively randomized studies have used different inclusion criteria [12, 29, 30]. The inclusion criteria proposed by ELSO are provided as an illustrative example [11] in Table 1.

**Table1:** Potential ECPR inclusion criteria, as suggested by ELSO [11]

Go criteria for ECPR
✓ Age < 70 years
✓ Witnessed cardiac arrest
✓ Time from arrest to CCPR ("no-flow interval") < 5 min (i.e., bystander CPR)
✓ Initial cardiac rhythm of ventricular fibrillation/pulseless ventricular tachycardia/pulseless electrical activity
✓ Time from arrest to initiation of ECMO flow ("low-flow interval") < 60 min
✓ End-tidal carbon dioxide (ETCO_2_) > 10 mmHg (1.3 kPa) during CCPR prior to ECMO
✓ Intermittent ROSC or recurrent ventricular fibrillation
✓ Presence of "signs of life" during CCPR may predict survival
✓ Absence of previously known life-limiting comorbidities (e.g., end-stage heart failure, chronic obstructive pulmonary disease, end-stage renal failure, liver failure, terminal illness) and alignment with the patient's care goals
✓ No known significant aortic valve incompetence (> mild aortic valve incompetence should be ruled out)

Survival after ECPR is influenced not only by the duration of resuscitation and the circumstances of the event, but also significantly by the age of the patient and pre-existing medical conditions. Therefore, elderly patients, those with pre-existing severe organ damage, or those with uncontrolled cancer are generally not considered for ECPR. In a recent investigation utilizing data from the global Extracorporeal Life Support Organization registry, a substantial elevation in mortality risk became evident starting at the age of 40, a departure from the expected timing. This underscores the challenges associated with patient selection, even within categories traditionally presumed to be straightforward to delineate (10.1007/s00134-023-07199-1).

The decision to pursue ECPR often relies on limited initial data, which may later turn out to be invalid, all within a time-sensitive and emotionally charged context.

### Low-flow

**Fig. 1:**
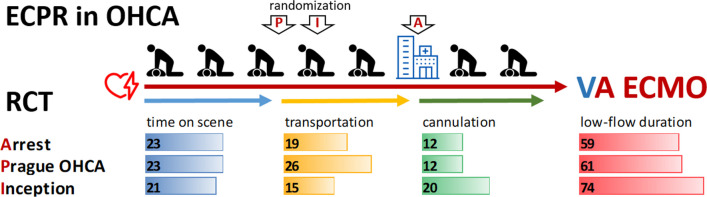
Low-flow in ECPR. Time under resuscitation (low-flow) observed in randomized controlled trials (RCTs) are given. Three RCTs examined the role of extracorporeal cardiopulmonary resuscitation (ECPR) in patients following out-of-hospital cardiac arrest (OHCA). Time plays a crucial role in ECPR, as prognosis strongly correlates negatively with low-flow duration. The three primary intervals in refractory OHCA—time spent on the scene, during transportation, and for cannulation for ECMO—are presented. Delays not attributed to one of these three main aspects of ECPR (time on scene, transportation, and cannulation) are not shown. It is worth noting that the 'Arrest' [30] and 'Prague OHCA' [29] trials both were single-center trials, while the 'Inception' [12] trial was multi-centered

The likelihood of good neurological survival diminishes rapidly during CCPR [5]. In ECPR, survival likewise declines along the duration of prior CCPR, but survival rates are higher (up to 30% at 20 min and 10–15% at 60 min) [7, 44]. Low-flow duration is arguably the most critical determinants of outcome, showing an almost linear relationship [7, 44]. This led to the commonly accepted paradigm that ECPR should be performed early, best within the “golden hour”, to reduce low-flow [7, 45, 46] and improve outcomes. Many steps are typically required between collapse and ECMO flow; see Fig. 1. Recognizing the importance of short low-flow, the focus of effective ECPR programs is on refining the process towards minimizing low-flow. Cannulation itself takes 10–15 min for well-trained teams [29, 30]. Such fast cannulations can only be achieved in an environment optimized for ECPR. In the recently published Inception study, notable for its high real-world relevance, median interval from hospital arrival to start of cannulation was 16 (interquartile rage IQR 11 to 22) minutes and a duration of cannulation of 20 (IQR 11 to 25) minutes [12]; see Fig. 1. The intricacies of each emergency system and hospital make it challenging to establish a universally applicable ECPR algorithm [47]. Considering the diverse operational frameworks of emergency systems and healthcare facilities across different countries and regions, localized efforts are essential. Analogous to the time benchmarks set for STEMI (ST-elevation myocardial infarction), where the interval from door to balloon insertion serves as a quality control [48, 49], and a similar principle should be applied to ECPR.

### Timing of ECPR

**Fig. 2:**
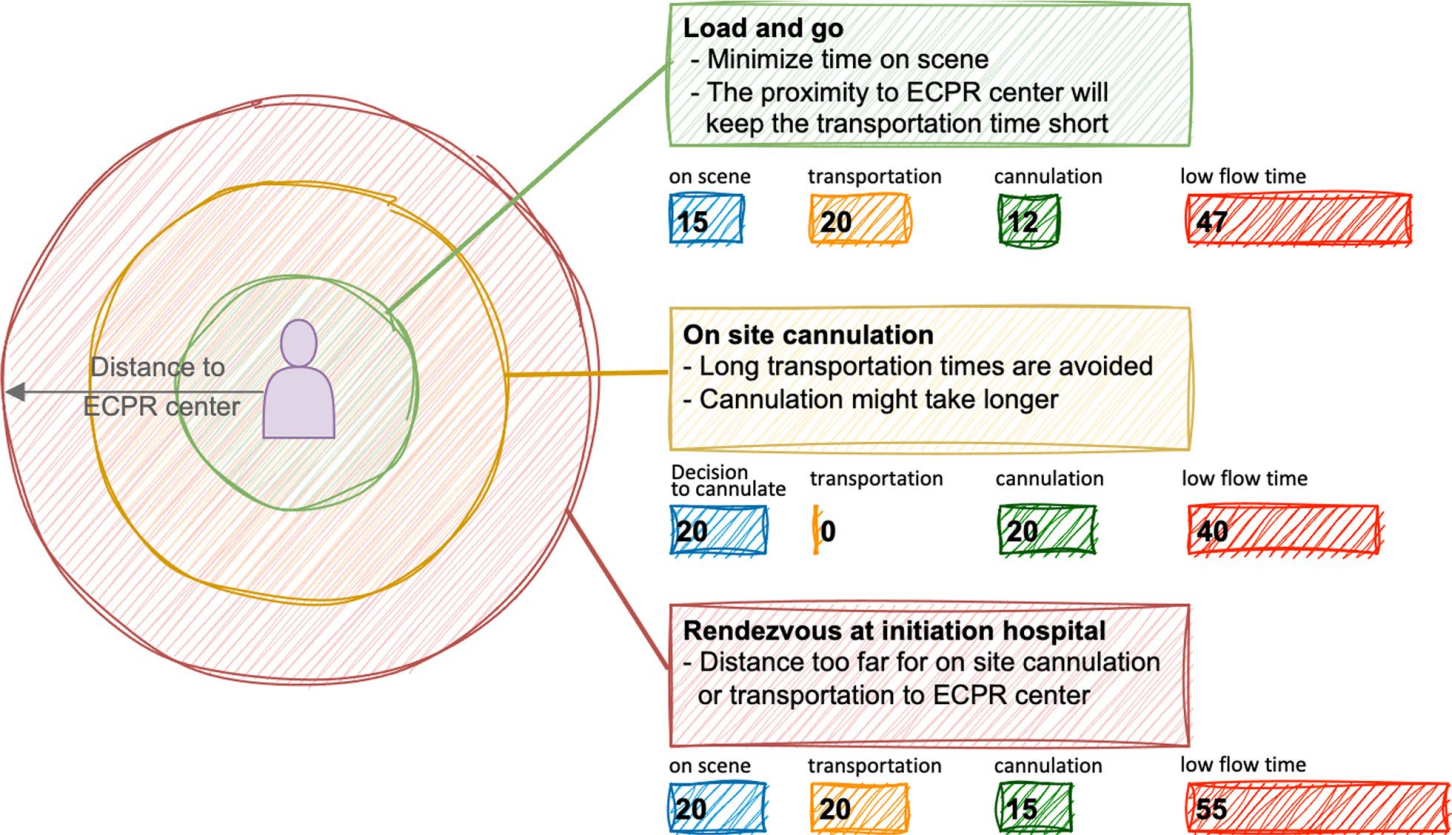
ECPR scenarios and distance of OHCA to the ECPR center. Potential scenarios for implementation of extracorporeal resuscitation (ECPR) in relation to the proximity of the place of out-of hospital cardiac arrest (OHCA) the ECPR center. Main aim is to minimize low-flow time. In the 'load and go' scenario, OHCA occurs in close proximity to the ECPR center. The victim is rapidly transported as soon as ECPR is designated as the treatment goal while the ECPR team assembles. The ‘on-site cannulation’ may save time when there is a significant expected transportation time. The ECPR team is alerted when an OHCA patient who qualifies for ECPR is identified and the team is transported to the site as quickly as possible. Although there is no transportation time until cannulation, the cannulation process itself may be challenging due to the unusual conditions. In more remote areas, a ‘rendezvous at initiation hospitals’, following the 'Minnesota model [107]', may be the optimal choice. Patients and the ECPR team convene at these dedicated hospitals, staffed with trained personnel. In all scenarios, it is theoretically possible to achieve ECPR cannulation and extracorporeal membrane oxygenation (ECMO) flow within a low-flow time of less than 60 min. Times given in this figure are estimates and not derived from clinical trials

**Fig. 3:**
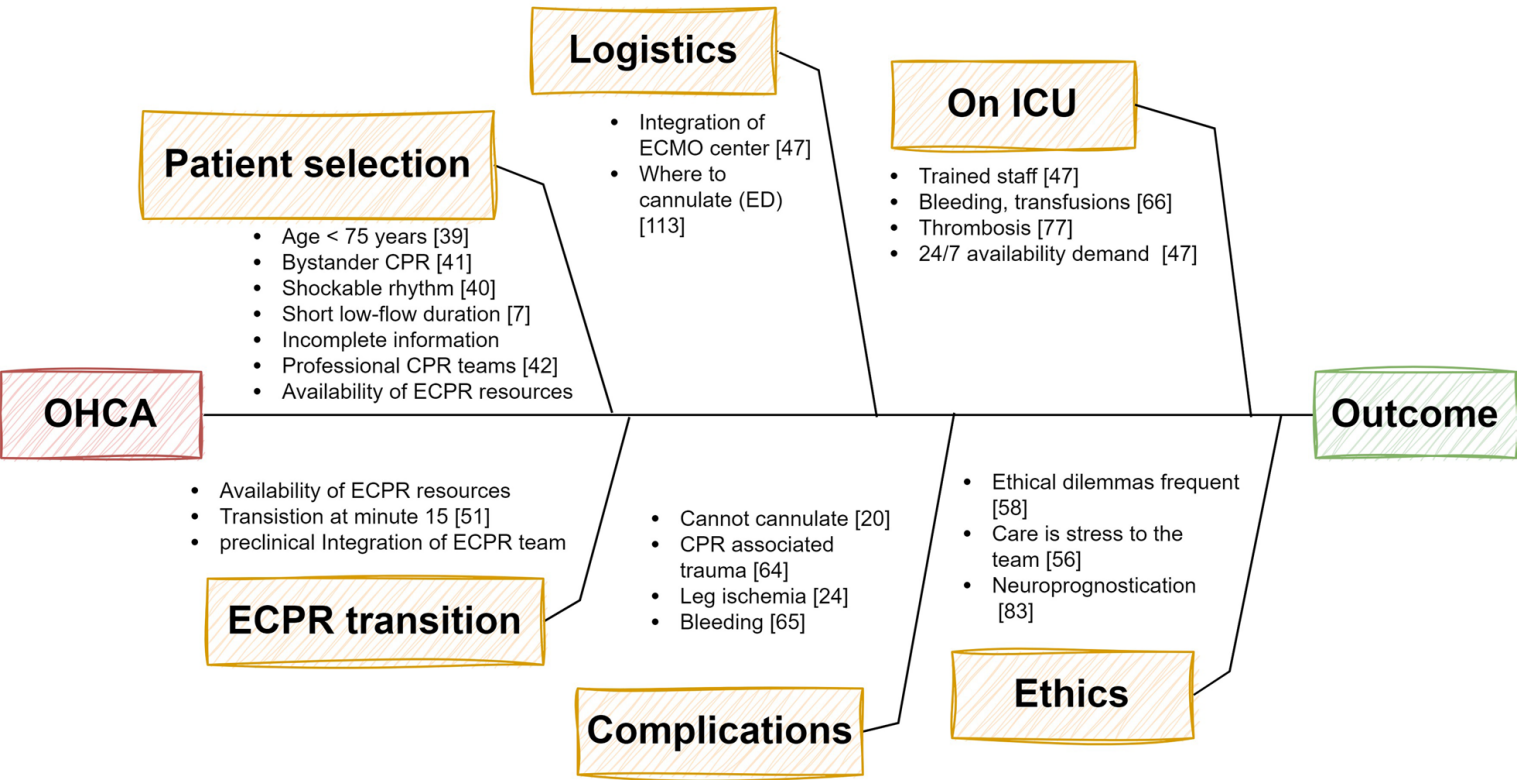
Challenges in ECPR, an excerpt. Extracorporeal resuscitation (ECPR) must seamlessly integrate into a highly complex scenario. Patient-related factors, various stakeholders, and institutional variables all influence the outcome. Numerous factors must be continually addressed and adapted to ensure a streamlined process. In addition to the aspects outlined here, many more may be significant, depending on local standards and patient pathways

The reciprocal correlation between outcome and duration on CCPR (low-flow duration) has been discussed earlier [7, 45, 46]. Cutting down on low-flow duration by beginning cannulation for ECPR earlier therefore seems intuitive. Doing ECPR too early, however, carries the risk that patients without refractory arrest would undergo an unnecessary and highly invasive treatment [12, 50]. Numerous efforts have been undertaken to identify the optimal transition time point from CCPR to ECPR [51]. Different emergency medical service systems are employed across international contexts. While some regions prioritize a "stay-and-play" approach, others emphasize "scoop-and-run" strategies; see Fig. 2. Data from the US showed that there is no benefit in a "stay-and-play" strategy beyond 15–20 min of CCPR [52]. However, survival improves when transporting the patient while resuscitation continues [52]. These observations align with studies suggesting a 12-min threshold for transitioning from CCPR to ECPR [53]. Establishing a swift transition from conventional to ECPR requires overcoming logistical challenges in order to minimize low-flow duration; see Fig. 3.

## On the ICU

The care for patients after ECPR is resource-intensive, incurring high costs and requiring substantial personnel involvement [54]. Many patients die within the first days after initiation of ECMO [12]. The mortality of up to 70% in the ELSO registry [3] despite ECPR can be particularly burdensome for attending physicians and nursing staff [55]. Consideration should also be given to the stress experienced by the patients' relatives [56]. The initial care on ICU includes standard post-resuscitation care [57], ECMO maintenance, and the prevention and treatment of complications [8]. Crafting a patient-centered, individualized therapy for the critically ill ECPR cohort presents a formidable challenge, even to experienced teams. Ethical issues are commonly reported in context of ECMO therapy [55, 58]. In depth discussion of ICU treatment is beyond the scope of this review.

ECPR is currently not a routine procedure but rather a last-resort treatment option for selected patients in situations where ECPR is available. Generally, ECMO therapy is used as a bridge to recovery when there is hope or expectation for the patient's improvement or, in cases of irreversible organ failure, as a bridge to organ transplantation or implantation of a durable left ventricular assist device (LVAD) [58].

After ECPR, about a third of patients die of anoxic brain injury [59]. Organ donation is possible in cases of brain death yielding the same results as conventional organ donation [60]. In some countries, organ donation is feasible after circulatory death (donation after cardiocirculatory determination of death, DCDD) [61, 62]. In such cases, organ retrieval can occur after cessation of life-sustaining ECMO support for a defined period (typically 5–15 min), during which cardiac arrest persists [63].

## Complications

ECPR is a highly invasive intervention. Besides complications associated with CCPR, such as fractures, tube misplacements, hemo- and pneumothorax, there are specific short-term complications unique to ECPR. These include access-site bleedings and cannula misplacements, primarily arising from the cannulation process [64]. Other typical complications will be discussed below.

### Coagulation

During ECPR, attention is crucial for thrombotic complications, hemolysis, and bleeding events [55, 65]. These issues arise from disturbances in coagulation due to patient blood contact with ECMO surfaces or mechanical stress [8, 66]. Undoubtedly, this is one of the most underestimated complications of ECMO therapy, which demands persistent vigilance and appropriate allocation of resources [67].

### Ischemia

The arterial cannula may occlude the lumen of the femoral artery in which it is inserted, leading to distal ischemia [68]. To avoid ischemic damage in this perfusion area, an additional cannula should be introduced antegradely into the femoral artery (typically the common femoral artery or superficial femoral artery) distal to the puncture site of the arterial cannula and connected to the returning ECMO cannula [69]. This way, oxygen-rich blood is directed into the peripheral blood vessels of the lower limbs. If routinely use of an antegrade leg perfusion cannula improves outcome is debated [70, 71] as there the inherit risk of access-site bleeding. Recent data suggest that bilateral cannulation, with arterial and venous cannulae placed on opposite sides, may lead to reduced complications, including lower rates of bleeding, decreased need for vessel repair, and a decrease in in-hospital mortality [72]. Selective perfusion: inserting the arterial ECMO cannula into the common femoral artery results in a non-physiological reverse laminar blood flow. The Harlequin syndrome is characterized by hypoxia in the upper half of the body despite sufficient oxygenation in the lower half [71]. In the context of V-A ECMO support during ECPR, poorly oxygenated blood from the lungs can enter the left ventricle and then flow into the aorta due to lung failure [73]. Inadequate oxygen supply can lead to ischemic brain damage or ischemic myocardium. The location of this watershed depends on volume status, cardiac ejection performance, and the blood flow rate of the ECMO system. Determining the exact location of the watershed is often challenging in clinical practice. To monitor cerebral oxygenation, pulse oximetry of the right upper extremity should be performed to exclude cerebral hypoxia. Additionally, near-infrared spectroscopy (NIRS) can be used to assess cerebral tissue oxygenation and provide early indications of insufficient cerebral oxygen supply [74].

### Cardiac function

Contemporary post-resuscitation guidelines are primarily derived from studies involving non-ECMO patients [57]. Consequently, hemodynamic management typically emphasizes traditional parameters including blood pressure, venous saturation, and lactate levels, rather than blood flow. V-A ECMO however primarily supports circulation by adding blood flow of up to 6 l/min to the native cardiac output. Quantifying the native cardiac output can be challenging as several specific points in hemodynamic monitoring of patients on V-A ECMO have to be considered [75]. Furthermore, by draining blood, the preload of the right ventricle might be reduced, and the endogenous cardiac output might decrease. The high blood flow and pressure in the aorta increases the afterload of the left ventricle along the mean arterial pressure. In cases of cardiac failure, the left ventricular function may be impaired to the extent that it cannot sustain adequate cardiac output under these conditions (reduced preload, preserved afterload), leading to pulmonary congestion and in case of stasis intra-cardiac thrombosis [76]. To counteract this, measures for left ventricular unloading should be considered whenever intrinsic cardiac output is deemed insufficient. The most common methods used beyond the application of inotropic or inodilatory medication for adjusting afterload for this purpose are the intra-aortic balloon pump (IABP) and the Impella device [77, 78]. Indications for these interventions are challenging, and there is currently no definitive data on the optimal timing and type of procedure, necessitating careful evaluation on a case-by-case basis. The recruiting Unload-ECMO trial might shed light on this field [78]. In the context of the previously described Harlequin syndrome and concomitant lunge failure, it may be necessary in individual cases to expand the V-A ECMO system to a veno-venoarterial (V-VA) system before implementing a left ventricular unloading device to ensure sufficient oxygenation of the blood in the left ventricle.

### Postresuscitation syndrome

In addition to the above-discussed organ systems, all the other organs have been subjected to an ischemia–reperfusion injury leading to multi-organ failure [79, 80]. The whole-body ischemia syndrome [81] is heterogeneous and requires a patient-centered symptomatic therapy according to the presentation [82]. Organs that typically need support are the lungs [83], vasoplegia [84], and the kidneys [85].

### Animal data

Animal ECPR studies are rare. A scoping review published 2023 by Ijuin and coauthors identified only 37 animal studies [86] in the context of ECPR. Over 90% of studies use a pig model for ECPR [87–95]. Ventricular fibrillation is induced by electrostimulation in over 70% of studies followed by a normothermic no-flow period of a median of 10 min [86]. Few studies use longer no-flow durations to up to 20 min [90, 91]. Only half of the animals are resuscitated before ECMO support [86]. These facts highlight the fundamental differences between the available animal model (young healthy animals with induced ventricular fibrillation) and the human ECPR reality (where patients are old, have a persistent cause of collapse, are resuscitated immediately after collapse and often cooled as soon as available [29, 96, 97]). Due to the long no-flow durations, those animals would not be considered candidates for ECPR according to current guidelines [11, 37]. A further problem with currently available animal data is that neurological outcome is rarely used as primary outcome [86, 93] opposed to human studies [12, 29, 30]. These facts need to be considered when bringing encouraging results from bench [87] to bedside.

## Clinical evidence

The first published randomized trial on ECPR was the ARREST trial published 2020 demonstrating a striking 43% good neurological survival (CPC 1–2 after 6 months) in the ECPR group opposed to 0% in the no-ECPR group [30]; see Table 2. At first glance, this seems plausible, since survival strongly declines with longer low-flow durations after OHCA [53, 98, 99], and IHCA [100]. Importantly, however, mortality without ECPR in selected patients (with witnessed arrest, immediate CCPR, younger age, shockable rhythm, treatable cause of arrest, etc.) even with longer low-flow durations is not 100% [53, 98, 99]. It is no coincidence that predictors of better outcome in CCPR (see above) are considered go-criteria for ECPR [11, 37]. Two larger randomized trials including well-selected patients could not demonstrate superiority of ECPR [12, 29]. While data from propensity score-matched registries reported conflicting results [101–103], newer meta-analysis of the randomized trials suggest improved survival [50, 104–106]; see Table 3. Ultimately, more data will be needed to prove a benefit of ECPR and to identify patients most likely to profit.

**Table 2:** Randomized data on ECPR

First author	Citation	Setting	Cannulation site	Patients in ECPR group	Hospital survival in ECPR	CPC 1–2 (6 months) in ECPR
Yannopoulos et al. 2020	[30]	OHCA	Hospital	15 (vs. 15)	43% (vs. 7%)	40% (vs. 0%)
Hsu et al. 2021	[108]	OHCA	Hospital	12 (vs. 3)	0% (vs. 33%)	0% (vs 0%)
Behlolavek et al. 2022	[29]	OHCA	Hospital	124 (vs. 132)	32% (vs. 23%)	32% (vs. 23%)
Suverein et al. 2023	[12]	OHCA	Hospital	70 (vs. 64)	20% (vs. 20%)	20% (vs. 16%)

**Table 3:** Pooled registry data on ECPR

First author	Citation	Patients	Setting	Data derived	Hospital survival in ECPR (%)	CPC 1–2 at maximum follow up
Richardson et al. 2017	[109]	1796	IHAC and OHCA	ELSO Registry	29	n.a
D’Arrigo et al. 2018	[110]	856	IHCA	Meta-analysis	38	32%
Inoue et al. 2022	[44]	1644	OHCA	Multi center registry, Japan	27	14%
Downing et al. 2022	[111]	1287	OHCA	Meta-analysis	24	18%
Kruit et al. 2023	[112]	222	Prehospital ECPR	Meta-analysis	23	n.a

## Conclusion

ECPR is invasive and resource intense. Data suggesting a survival benefit in patients after OHCA and ECPR derive from retrospective registries and meta-analyses. In order to improve outcomes, ECPR teams have to be embedded into local emergency systems and refined towards a reduction in low-flow.

## Data Availability

Not applicable.
